# EGFR-Driven Mutation in Non-Small-Cell Lung Cancer (NSCLC) Influences the Features and Outcome of Brain Metastases

**DOI:** 10.3390/jcm12103372

**Published:** 2023-05-09

**Authors:** Daniele Armocida, Alessandro Pesce, Mauro Palmieri, Fabio Cofano, Giuseppe Palmieri, Paola Cassoni, Carla Letizia Busceti, Francesca Biagioni, Diego Garbossa, Francesco Fornai, Antonio Santoro, Alessandro Frati

**Affiliations:** 1 Human Neurosciences Department, Neurosurgery Division, “Sapienza” University, 00161 Rome, RM, Italy; danielearmocida@yahoo.it (D.A.); mauro.palmieri@uniroma1.it (M.P.); antonio.santoro@uniroma1.it (A.S.); 2 IRCCS “Neuromed”, 86077 Pozzilli, IS, Italy; carla.busceti@neuromed.it (C.L.B.); francesca.biagioni@neuromed.it (F.B.); francesco.fornai@neuromed.it (F.F.); 3 Neurosurgery Unit, “Santa Maria Goretti” University Hospital, 04100 Latina, LT, Italy; ale_pesce83@yahoo.it; 4 Neurosurgery Unit, Department of Neuroscience “Rita Levi Montalcini”, University of Turin, 10126 Turin, TO, Italy; fabio.cofano@gmail.com (F.C.); giuseppe.palmieri@unito.it (G.P.); diego.garbossa@unito.it (D.G.); 5 Pathology Unit, Department of Medical Sciences, University of Turin, 10126 Turin, TO, Italy; paola.cassoni@unito.it

**Keywords:** brain metastases, lung cancer, NSCLC, EGFR, ALK, brain tumor

## Abstract

**Background:** Brain metastases (BMs) is one of the most frequent metastatic sites for non-small-cell lung cancer (NSCLC). It is a matter of debate whether EGFR mutation in the primary tumor may be a marker for the disease course, prognosis, and diagnostic imaging of BMs, comparable to that described for primary brain tumors, such as glioblastoma (GB). This issue was investigated in the present research manuscript. **Methods:** We performed a retrospective study to identify the relevance of EGFR mutations and prognostic factors for diagnostic imaging, survival, and disease course within a cohort of patients affected by NSCLC-BMs. Imaging was carried out using MRI at various time intervals. The disease course was assessed using a neurological exam carried out at three-month intervals. The survival was expressed from surgical intervention. **Results:** The patient cohort consisted of 81 patients. The overall survival of the cohort was 15 ± 1.7 months. EGFR mutation and ALK expression did not differ significantly for age, gender, and gross morphology of the BM. Contrariwise, the EGFR mutation was significantly associated with MRI concerning the occurrence of greater tumor (22.38 ± 21.35 cm^3^ versus 7.68 ± 6.44 cm^3^, *p* = 0.046) and edema volume (72.44 ± 60.71 cm^3^ versus 31.92 cm^3^, *p* = 0.028). In turn, the occurrence of MRI abnormalities was related to neurological symptoms assessed using the Karnofsky performance status and mostly depended on tumor-related edema (*p* = 0.048). However, the highest significant correlation was observed between EGFR mutation and the occurrence of seizures as the clinical onset of the neoplasm (*p* = 0.004). **Conclusions:** The presence of EGFR mutations significantly correlates with greater edema and mostly a higher seizure incidence of BMs from NSCLC. In contrast, EGFR mutations do not affect the patient’s survival, the disease course, and focal neurological symptoms but seizures. This contrasts with the significance of EGFR in the course and prognosis of the primary tumor (NSCLC).

## 1. Introduction

Brain metastases (BM) are the most common intracranial tumors in adults and significantly affect lethality. Roughly, 40% of patients with malignancies develop intracranial metastases during the disease course. Lung cancer is the neoplasm, which leads to the highest percentage of brain metastases [1,2,3]. Among various lung cancers, non-small cell lung cancer (NSCLC) is the leading cause of BMs and represents the most frequent cancer-related death worldwide [1]. Approximately 5–30% of all BMs derive from NSCLC [4,5]. Over 40% of patients carrying an early diagnosis of lung cancer develop BMs during the disease course [1,2,6,7], which significantly worsens the life expectancy and the quality of life (QoL) [8].

The increase in BMs, which was registered in the last decades, is likely due to prolonged life duration, which is achieved in the general population affected by neoplasm [9,10], and mainly due to advances in the treatment of primary cancer, and an earlier diagnosis of BM due to an improvement in neuroimaging techniques [11]. However, despite current standard treatments represented by microsurgical resection, focal fractionated radiotherapy, stereotactic radiosurgery, and BM from NSCLC continues to be associated with a poor prognosis [12,13,14,15,16]. Therefore, at present, intense research activity is dedicated to unravelling the key molecular targets of NSCLC to develop novel therapeutic strategies to treat NSCLC-derived BMs. A key molecule characterizing NSCLC is epidermal growth factor receptor (EGFR), along with anaplastic lymphokinase (ALK), and PD-L1 [17]. In fact, it has been observed that the incidence of BM is higher in patients with ALK fusions [18,19,20] and EGFR mutations [18,19,20,21,22]. In detail, EGFR positivity or ALK-1 rearrangements (which are mutually exclusive in their occurrence) are associated with the worsening of tumor progression. The ability to identify these targets prompted specific therapies that modified the prognosis of the primary tumor. Therefore, at present, EGFR positivity represents a therapeutic advantage to delay tumor progression by using specific tyrosine-kinase inhibitors, which improves survival and reduces the relapse of lung cancer, thus leading to a better prognosis [23,24], even considering that EGFR positivity is often associated with PD-L1 negativity [25]. When considering the therapeutic development achieved via treating EGFR-positive primary NSCLC, one may argue that the same outcome may apply to NSCLC-derived BMs. Unfortunately, NSCLC-derived EGFR-positive metastasis does not respond positively to the specific treatment [26,27]. To understand the significance of EGFR positivity for the natural course of NSLC-derived BMs, we carried out the present study.

With this aim, we retrospectively analyzed a consecutive series of patients who had resection surgery of NSCLC-derived BMs from January 2015 to January 2019 at the Department of Neurosurgery of Policlinico Umberto I of Rome (Italy) and Hospital Molinette of Turin (Italy). In this study, we retrospectively identify the significance of the occurrence of EGFR mutations by assessing life expectancy, neurological symptoms in the disease course, and neuroimaging (to assess neoplasm and edema volume measured on FLAIR sequences) in a cohort of 81 surgically treated patients.

## 2. Materials and Methods

### 2.1. Participants and Eligibility

All the patients included in the final cohort meet the following inclusion criteria:

1. A preoperative KPS scale score >50%.
2. An estimated overall survival of >3 months (according to the radiation therapy oncology group and the grade prognostic assessment rankings) [28,29]
3. The estimated target of the surgical procedure was the gross-total, near-total, or sub-total resection of the lesions: no biopsies were included. We included those patients where complete surgical resection could be guaranteed by pre-operative planning, thus excluding cases with multiple deep-site metastases that could not be surgically treated by definition. Patients with sub-centimetric heteroplastic lesions were included after dedicated conformational radiotherapy regimens.
4. The molecular analysis of EGFR mutations was carried out in the brain metastases in addition to the primary NSCLC.
5. Only patients who may undergo post-surgical adjuvant chemo-radiotherapy and a follow-up program were included.
6. Patients were included if they received standard conformational planning with a linear accelerator (LINAC).
7. Once the progression of the disease was noticed, the patient and the relevant imaging were referred again to our attention to evaluate the feasibility of a second surgery or to address the patient to the second line of adjuvant treatment.

All patients underwent a general medical, neurological, and oncological evaluation at admission. For all patients, we recorded gender; age; peri- and post-operative KPS; clinical presentation; survival; antiepileptic prophylaxis and treatment; the incidence of postoperative seizures; and tumor- and surgery-related variables: number, location and side of the lesions, tumor and edema volume, morphology, the onset of the primary tumor, and molecular profile (EGFR, ALK, and PD-L1).

In particular, the specimens used in this study were examined for EGFR. DNA mutations in EGFR were detected using polymerase chain reaction (PCR) to identify mutations within exons 19 and 21. Immunohistochemistry for CDX-2, CK7, CK20, TTF-1, and Napsin-A was routinely carried out. Overall survival (OS) was recorded in months; it was measured from the date of diagnosis to the fatality considering the last contact when alive. Clinical information was obtained using the digital institutional database. A particular focus was centered on the performance status expressed as KPS results, which were used as dichotomy data (either more or less than score 70, KPS). This score was chosen since it is critical for a patient’s survival when BM are present [13,30,31,32]. KPS was recorded before surgery at the time of diagnosis and it was repeated 30 days after surgery (early post-operative evaluation and it was further recorded at the end of the adjuvant treatment, the last outpatient evaluation).

### 2.2. Preoperative and Operative Protocol

All patients received a pre-operative brain MRI scan, including a 3 Tesla volumetric study with the following sequences: T2w, fluid-attenuated inversion recovery (FLAIR), and isotropic volumetric T1-weighted magnetization-prepared rapid acquisition gradient echo (MPRAGE) before and after intravenous administration of paramagnetic contrast agent; diffusion tensor sequences (DTI) with 3D tractography and functional MRI (fMRI) completed our protocol for what concerns lesions affecting eloquent locations. The volume of the contrast-enhancing lesion was calculated by drawing a region of interest (ROI) in a volumetric enhancing post-contrast study weighted in T1 (a multi-voxel study), conforming to the margins of the contrast-enhancing lesion. In contrast, the volume of edema was measured by drawing an ROI in a FLAIR-weighted research, from which the previously calculated lesion was subtracted. The study was carried out using the Horos Dicom Viewer (v 3.36, opensource software, Pixmeo SARL, Bernex, Switzerland; https://horosproject.org/) [33]. Moreover, we routinely performed total-body sodium-enhanced CT and bone scintigraphy to complete the oncology staging protocol.

In a standard neurosurgical theatre, all the procedures were performed with an infrared-based Neuro-navigator (Brainlab, Kick^®^ Purely Navigation), with a standard operative microscope. During the first post-operative day, as routine, the patients underwent a CT scan to exclude major complications and a volumetric brain MRI scan to evaluate the EOR. For both groups, in the case of lesions placed within non-eloquent areas, a standard total intravenous anesthesia protocol with Propofol (1 mg/kg) and Remifentanil (0.5 mg/kg/min) was applied. For lesions involving the sensory-motor and language-related cortex, a standard full awake surgery protocol was routinely performed with the aid of intraoperative neuro-monitoring realized using bi- and monopolar stimulating probes, respectively, for the cortical and sub-cortical mapping. No muscle relaxants were administered when intra-operative neuromonitoring or no awake surgery was performed [34]. During surgery, tumor excision was arrested when:

1. Despite a directly visualized or navigation-proven remnant, neuromonitoring or intraoperative neuropsychological testing outlined a risk for postoperative sensory-motor damage,
2. The white matter appeared free of disease in each aspect of the surgical cavity.

### 2.3. Data Sources and Quantitative Variables

The extent of resection (EOR) was determined by comparing the MR images obtained before surgery and the first early MRI after surgery, following the RANO criteria [35]. EOR was coded in a 3-step ordinal variable as reported elsewhere [11]: gross-total resection (GTR) <2 mm^3^ residual lesions; near-total resection (NTR) (≧2 to <5 mm^3^), and sub-total resection (STR) (≧5 mm^3^).

In the case of GTR, “tumor progression” was defined as the first MRI scan demonstrating the presence of pathologically enhancing tissue characterized using an MRI pattern (mainly relying on perfusion-weighted imaging) inconsistent with a cerebral radiation injury (which is, in fact, a “pseudo-progression”). In incomplete resections (NTR/STR), a volumetric increase in the residual disease detected at the first postoperative MRI scan was considered as disease progression. A close-range dedicated neuro-imaging follow-up program was routinely performed at our institution. This program included:

A standard early (maximum 24 h after surgery) postoperative volumetric brain MRI; at approximately one month from surgery (25–35 days), a volumetric brain MRI scan was repeated for a first step follow-up control and information for the radiation treatment planning; a volumetric brain MRI scan was performed every three months at the end of irradiation; and we performed a complete medical and neurological outpatient re-evaluation at every radiological reevaluation.

### 2.4. Size, Statistics, and Potential Source of Bias

The study size was determined based on the selection of the inclusion criteria. The sample was analyzed with SPSS v18 (SPSS Inc., released 2009, PASW Statistics for Windows, Version 18.0, Chicago, IL, USA) to outline potential correlations between variables under investigation. Comparisons between nominal variables were carried out using the Chi^2^ test. EOR, OS, PFS mean, edema, lesion volume, and their correlations with EGFR mutations were compared with one-way and multivariate ANOVA analysis and contrast analysis and post-hoc tests. Kaplan–Meier survival analysis was carried out. Continuous variable correlations were investigated using Pearson’s bivariate correlation. The threshold of statistical significance was considered *p* < 0.05.

### 2.5. Ethical Issue

The Institutional Review Board approved the informed consent at our Institution (IRB 6168 Prot. 0935/2020). Before the surgical procedure, all the patients gave informed written explicit consent after appropriate information. The data reported in the study have been completely anonymized. No treatment randomization was carried out. This study is perfectly consistent with the Helsinki Declaration of Human Rights in Medical Research.

## 3. Results

In a period ranging from January 2015 to January 2019, a total of 81 patients suffering from NSCLC brain metastases have been operated on in our Neurosurgical Departments. A total of 27 patients were female (33.3%), and 54 were male (66.7%) with a 1:2 ratio. The average age of the cohort was 62.1 years ± 10.9. In this cohort, brain metastasis favored frontal (32 patients, 39.5%) and cerebellar (18 patients, 22.2%) localization; in general, the lesions were more commonly found in the supratentorial compartment (77.8%) with no infratentorial involvement but the cerebellum. The frontal placement is statistically significant (*p* < 0.001). Thirty-six patients had a right lesion, 43 left, while just 2 involved the midline. No statistically significant side-specificity was evident. The tumor morphology was mostly solid and compact (61.7%), whereas BMs presented as cystic lesions in 19.7% of cases. The average volume of the lesions and perilesional edema were, respectively, 14.62 ± 18.5 cm^3^ and 54.21 ± 45.76 cm^3^. The diagnosis and clinical presentation were more commonly synchronous (60.5%) rather than metachronous and with sensory-motor dysfunction (41.9%) or with seizures (27.2%). In 59 cases (72.8%), a GTR was achieved. A total of 67 patients presented a preoperative KPS over 70 before surgery, whereas 73 had the same performance status at the 30th post-operative day re-evaluation (*p* = 0.001). The overall survival of the cohort was 15 ± 17 months (data reassumed in Table 1).

### 3.1. Volume and Edema

**Figure 1:**
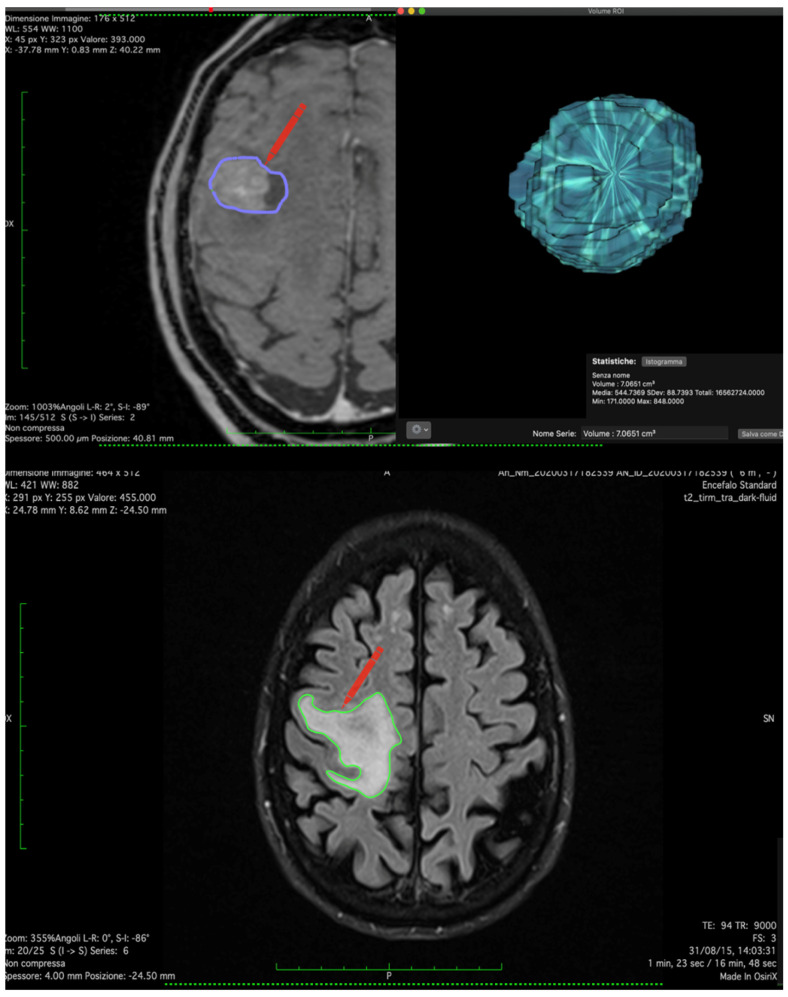
Figure shows tumor reconstruction using Horos software with volumetric calculation of contrast capturing lesion and peritumoral edema.

**Figure 2:**
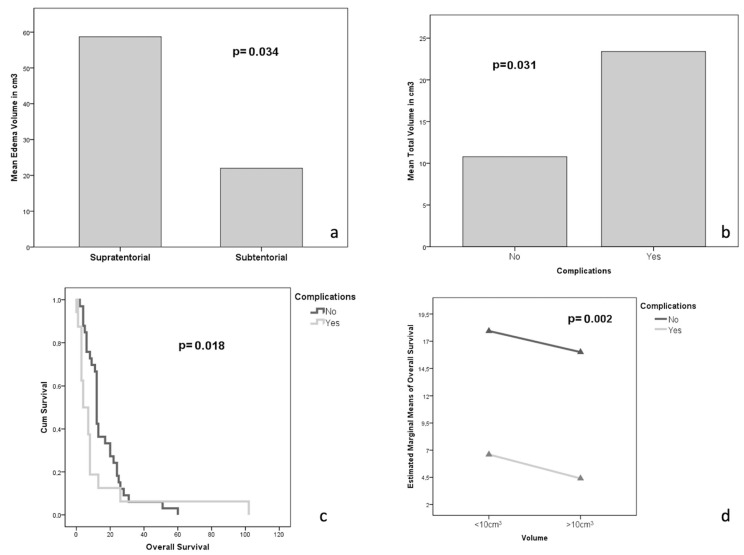
(**a**) One-way ANOVA analysis demonstrating the association between edema volume and the intracranial compartment. (**b**) One-way ANOVA analysis demonstrating the association between total volume and the incidence of complications. (**c**) Kaplan–Meier survival curve demonstrating the impact of complications on survival. (**d**) Multivariate ANOVA analysis demonstrating the association between complications and survival independently from the volume of the lesion.

**Figure 3:**
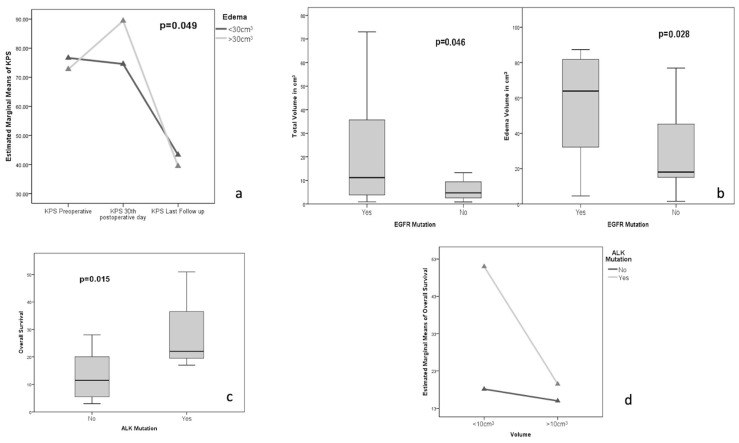
(**a**) Repeated measures ANOVA analysis demonstrating the impact of edema on the functional status (**b**) One-way ANOVA analysis demonstrating the association between EGFR mutation status and the lesion and edema volumes. (**c**) One-way ANOVA analysis demonstrating the association between ALK mutation status and Overall Survival. (**d**) Multivariate ANOVA analysis demonstrating the association between ALK mutation status and survival with respect to the volume of the lesion: the survival advantage disappears for the greater lesions.

Tumor volume and edema demonstrated a significant reciprocal association (r = 0.369, *p* = 0.010) (Figure 1), and the more significant edema was associated with supratentorial placement (*p* = 0.034, Figure 2a). The tumor-related edema demonstrates an association with neurological symptoms at the beginning of the disease (*p* = 0.048) rather than with the volume of the lesion per se (*p* = 0.891), outlining that the tumor-associated edema is more commonly responsible for the neurological symptoms rather than a greater tumor volume itself. Moreover, a greater tumor volume was associated with a higher incidence of complications (*p* = 0.031, Figure 2b), which, in turn, was also associated with significantly shorter survival (*p* = 0.018 Figure 2c). This finding is exciting when observing, on a multivariate ANOVA analysis, that complications, per se, negatively affect survival, independently of tumor volume (*p* = 0.002, Figure 2d). Furthermore, on a repeated measures ANOVA analysis, edema was demonstrated to play a statistically significant role (*p* = 0.049, Figure 3a) affecting the early post-operative period: patients with edema volume greater than 30 cm^3^ had a poorer outcome on post-operative day 30th at KPS when compared with pre-operative and late follow up time intervals.

### 3.2. EGFR-Related Parameters

In all cases, the presence of EGFR was confirmed both within the primary tumor and its BM. This is important since some cases may possess EGFR mutations in the primary tumor but not within its BM, while in the presence of an EGFR-positive BM, the primary tumor necessarily expresses EGFR as well. EGFR mutations were neither significantly associated with gender, age, nor with the shape and number of the BM (Table 2); in contrast, the size and peri-lesion edema were significantly associated with the EGFR mutations (for BM’s volume 22.38 ± 21.35 cm^3^ in EGFR expressing BMs versus 7.68 ± 8.44 cm^3^ in non-EGFR expressing BMs while for peri-lesion edema was 72.44 ± 60.71 cm^3^ versus 31.92 cm^3^; *p* = 0.046 and *p* = 0.028, respectively. Figure 3b). Remarkably, in our series, EGFR was not associated with any specific neurological symptoms apart from the incidence of pre-operative seizures (*p* = 0.004). EGFR was not associated with survival, while the expression of ALK, as previously reported [36], was strongly associated with survival. In fact, the cumulative survival of patients presenting ALK mutation was 30.0 ± 18.36 months compared with 12.88 ± 8.31 months in the wild-type ALK phenotype (*p* = 0.015, Figure 3c). ALK mutation was associated with better survival in patients harboring smaller lesions, possibly with smaller edema volumes. In great lesions (>10 cm^3^) with bigger edema (>30 cm^3^), the survival advantage of ALK mutation disappears (Figure 3d).

## 4. Discussion

So far, the current treatment of BMs is represented by RT (or radiosurgery) or microsurgery followed by RT [37,38]. Concerning NSCLC, treatment protocols have been radically changed by discovering molecular targets, such as EGFR and ALK, and the subsequent development of specific drugs aimed to block these receptors. However, their efficacy in patients affected by BMs is not fully understood because this group of patients is usually excluded from clinical studies, especially when neurologically symptomatic [8,39]. Therefore, a standard treatment schedule for these patients needs to be identified [40].

Recent findings suggest that driver mutations in NSCLC would be at least partly associated with the development of BMs in NSCLC. More specifically, EGFR mutations have been detected in 64% and 31% of patients with and without BMs, respectively, suggesting that brain metastases would be more frequent in tumors bearing EGFR mutations [41]. Recent evidence regarding anaplastic lymphoma kinase (ALK) translocations also indicates that they may predispose to brain metastasis formation [42].

Although ALK translocations might correlate with the development of BMs, they may represent a beneficial event for prognosis. Nevertheless, the association between ALK positivity and prognosis is widely debated [43]. Further studies are needed to establish a correlation between ALK mutation and better survival in patients with small brain lesions. Despite the occurrence of EGFR mutations that improve the prognosis of primary lung neoplasm [44], such an association could not be confirmed in these studies concerning EGFR-positive BMs. Further evidence is needed to confirm this finding and specific correlative studies should consider lesion volumes and amount of brain edema. In fact, as described in this study, the presence of larger volumes of edema is associated with a higher incidence of neurological symptoms. Treatment of both the primary neoplasms and the BMs is not contraindicated solely by a single BM, and complete resection of all diseases should be attempted whenever safe and feasible. Operative mortality and morbidity for this combined approach are low [45]. In fact, given the encouraging results in terms of survival, primary tumor resection and treatment (neurosurgical intervention or irradiation) for synchronous lung and brain lesions appear to be justified [15,32,33,34,46]. In our cohort, we defined two groups of patients harboring BMs: those with a single lesion synchronous with the primary tumor and those with a solitary lesion that develops months or years after successfully managing the primary tumor. Some authors observed that the synchronous presentation of lung cancer and BMs is a negative prognosis factor [14,17,47].

In the present study, several factors (functional status, general health conditions, morphological and histological features of the lesions, and prognostic indices) have been investigated to analyze their association with the risk of death at 12 weeks and at one year. Among these, only the KPS score (>70%) and RT application appear to be significant protective factors [47,48,49,50,51]. Moreover, morbidity and mortality rates have decreased significantly with improved neurosurgical techniques and perioperative care. Most single BMs are manageable by total resection, performed on 72.84% of the patients with low mortality and morbidity rates, in line with data reported in the literature [44]. Nevertheless, the surgical indication in debilitated patients with advanced systemic disease should be carefully considered because the morbidity and deaths in our study were primarily due to systemic and infectious complications.

In BMs without surgical indication, treatment options, such as SRS or SRS and whole-brain radiotherapy (WBRT), should be performed whenever feasible by tailoring the treatment protocol to both the patient’s and the diseases’ specificity. Prophylactic cranial irradiation (PCI) has been abandoned due to complications, such as cognitive disorders and neurological deficits [52]. This strengthens the need to develop accurate approaches to identify those patients affected by lung cancer-bearing an increased risk of developing BMs. In this scenario, the role of ALK and EGFR could be relevant in the immediate future [26,27,35].

The main limitation of the present paper is its retrospective nature. Moreover, the current investigation was conducted on a subset of BMs patients, which met the criteria for surgical indication, and who had relatively good functional status. Therefore, this may affect the general outcome of all the patients suffering from BMs from NSCLC of the present findings. Another potential bias is the limited availability of the ALK and EGFR status in the entire cohort (41 ALK and 37 EGFR-investigated patients). Nevertheless, the conclusions reported here are statistically significant, thus, providing exciting clues concerning the use of ALK and EGFR in patients’ stratification.

The occurrence of high correlation between EGFR mutations and seizure incidence may extend the significance of this study making EGFR more than a mere disease marker to disclose novel avenues in the pathophysiology of BMs and epilepsy. In fact, in a very recent paper, where patients affected by mesial temporal lobe sclerosis and limbic seizures were analyzed, abnormal EGFR signaling was measured [26], which poses a causal relationship between EGFR mutations in BMs, primary tumors, and seizure onset.

## 5. Conclusions

According to the results of the present study, the presence of EGFR mutations correlates with edema, volumes of the lesion, and a higher incidence of seizures, while no effects were noticed on prognosis. Contrariwise, the presence of ALK translocations in BMs deriving from NSCLC could be associated with a better prognosis. Given the dense scientific debate on the role of EGFR and ALK mutations in NSCLC, aimed studies on BMs derived from this specific family of lung cancer should be carried out to explore their impact on diagnosis and treatment prognosis.

## Figures and Tables

**Table 1:** Patient’s demographics.

	N = 81 Patients
Sex	Male N = 54–66.7%
	Female N = 27–33.3%
Age	62.1 years ± 10.9
KPS at admission	>70 = 67–82.7%
	<70 = 14–17.3%
GPA for 80 pts (1 missing datum)	3 = 4 pts
	2.5 = 14 pts
	2 = 16 pts
	1.5 = 22 pts
	1 = 22 pts
	0.5 = 2 pts
KPS after surgery (30 d)	>70 = 73–90.1%
	<70 = 9–11.1%
KPS at last Evaluation	>70 = 49–60.5%
	<70 = 32–39.5%
Dead 68/81 pts at 09/20	48 dead
	20 alive
Overall Survival	15 ± 1.7 months
Volume (cm^3^)	14.62 ± 18.5
Edema Volume (cm^3^)	54.21 ± 45.76
Periventricular	11 pts–15.1%
Location	Supratentorial = 63–77.8%
	Subtentorial = 18–22.2%
Major Lobe involved	Frontal 32 (39.5%)
	Temporal 5 (6.1%)
	Occipital 10 (12.34%)
	Parietal 16 (19.75%)
	Cerebellar 18 (22.22%)
Side	Left 43 (53.1%)
	Right 36 (44.4%)
	Midline 2 (2.47%)
Symptoms at onset	Seizures 22 (27.16%)
	Sensory-Motor Dysfunction 34 (41.9%)
	Asymptomatic (follow-up) 25 (30.8%)
Antiepileptic Profilaxis and Treatment	43 pts (53.1%)
Post-operative Seizure	25 pts (30.86%)
Surgical Resection	GTR = 59 (72.84%)
	STR = 22 (27.16%)
Morphology of Tumors	Solid = 50 (61.73%)
	Cystic = 16 (19.75%)
	Hemorragic = 8 (9.87%)
	Mixed = 7 (8.6%)
Onset	Synchronous = 49 pts (60.5%)
	Metachronous = 32 pts (39.5%)
Extracranial metastases	5 pts (6.2%)
*Immunohystochemical/molecular features*	
EGFR mutation	Expressed = 56.25%
	Not expressed = 43.75%
ALK mutation	Expressed = 17%
	Not expressed = 83%
PD-L1 expression with tumor proportion score (TPS) ≥1%	(TPS) ≥1% = 54% of pts
	Not Expressed = 46% of pts

Abbreviations: Karnofsky performance status (KPS), Graded Prognostic Assessment (GPA), Gross-total resection (GTR), Sub-total resection (STR), tumor proportion score (TPS).

**Table 2:** EGFR mutation groups analysis.

Total 81	EGFR +	EGFR −	*p*-Value
**N° of cases**	45	36	
**Age**	61.34 ± 11.11	63.1 ± 9	1
**Volume (cm^3^)**	22.38 ± 21.35	7.68 ± 6.44	0.046
**Edema volume (cm^3^)**	72.44 ± 60.71	31.92	0.028
**Clinical debut**			
Seizure	20	2	0.004
Sensory-Motor Dysfunction	16	18	1
Asymptomatic (follow-up)	9	16	0.41
**Morphology**			1
Hemorragic	25	21	
Cistic	7	9	
Solid	6	2	
Mixed	3	4	
**Overall Survival**	12 ± 4.3	16 ± 6.5	0.77

## Data Availability

Data are available on request to the corresponding author.
